# A Student’s Guide to Neural Circuit Tracing

**DOI:** 10.3389/fnins.2019.00897

**Published:** 2019-08-27

**Authors:** Christine Saleeba, Bowen Dempsey, Sheng Le, Ann Goodchild, Simon McMullan

**Affiliations:** ^1^Neurobiology of Vital Systems Node, Faculty of Medicine and Health Sciences, Macquarie University, Sydney, NSW, Australia; ^2^The School of Physiology, Pharmacology and Neuroscience, University of Bristol, Bristol, United Kingdom; ^3^CNRS, Hindbrain Integrative Neurobiology Laboratory, Neuroscience Paris-Saclay Institute (Neuro-PSI), Université Paris-Saclay, Gif-sur-Yvette, France

**Keywords:** neuroanatomy, viral tracers, anterograde tracer, retrograde tracers, synaptic contacts, connectome analysis

## Abstract

The mammalian nervous system is comprised of a seemingly infinitely complex network of specialized synaptic connections that coordinate the flow of information through it. The field of connectomics seeks to map the structure that underlies brain function at resolutions that range from the ultrastructural, which examines the organization of individual synapses that impinge upon a neuron, to the macroscopic, which examines gross connectivity between large brain regions. At the mesoscopic level, distant and local connections between neuronal populations are identified, providing insights into circuit-level architecture. Although neural tract tracing techniques have been available to experimental neuroscientists for many decades, considerable methodological advances have been made in the last 20 years due to synergies between the fields of molecular biology, virology, microscopy, computer science and genetics. As a consequence, investigators now enjoy an unprecedented toolbox of reagents that can be directed against selected subpopulations of neurons to identify their efferent and afferent connectomes. Unfortunately, the intersectional nature of this progress presents newcomers to the field with a daunting array of technologies that have emerged from disciplines they may not be familiar with. This review outlines the current state of mesoscale connectomic approaches, from data collection to analysis, written for the novice to this field. A brief history of neuroanatomy is followed by an assessment of the techniques used by contemporary neuroscientists to resolve mesoscale organization, such as conventional and viral tracers, and methods of selecting for sub-populations of neurons. We consider some weaknesses and bottlenecks of the most widely used approaches for the analysis and dissemination of tracing data and explore the trajectories that rapidly developing neuroanatomy technologies are likely to take.

## Introduction

The relationship between structure and function is a central theme in the field of biology. In the same way that deciphering the crystal structure of DNA propelled research toward the mechanics of inheritance in the 20th century (Watson and Crick, 1953), it is widely believed that elucidation of the structural architecture of the brain will fundamentally alter neuroscience in the 21st (Rubinov and Bullmore, 2013; Lerner et al., 2016; Mikula, 2016). Here, we review the approaches used by contemporary neuroscientists to identify connectivity patterns between components of neural circuits, the trajectories that rapidly developing neuroanatomy technologies are likely to take, and some bottlenecks that may hinder this mission.

It is unknown at what point in history the brain was first recognized as the control center for the body. Although sometimes attributed to Hippocrates and Galen around 2,000 years ago, the association between traumatic brain injury and distinct functional deficits was clearly understood by Egyptian physicians 30 centuries before that (Stiefel et al., 2006), and archeological evidence indicates widespread neurosurgical practice in diverse cultures since time immemorial (Andrushko and Verano, 2008, reviewed by Kshettry et al., 2007; Moghaddam et al., 2015). What is clear is that the association between brain structure and function is a relatively recent realization: this consensus was only reached in the late 1800s after nearly 100 years of disagreement between those who, like influential French physiologist and child prodigy Marie-Jean-Pierre Flourens, believed that the brain, like the mind, was indivisible (Pearce, 2009), and those like Frantz Joseph Gall who proposed that the brain is composed of distinct functional compartments, and that the relative contribution of each is to some extent independent from the others [see Ferrier (1884) for contemporaneous review].^1^

At the same time that Broca and others were cementing the idea of cerebral localization at a macroscopic scale, the first histologists were using the microscope to discover the delicate structure of neural tissue and developing theories about the cellular basis of brain function. The “neuron theory” developed by Ramon y Cajal, Waldeyer-Hartz and others argued that a particular cell type, the neuron, was the functional unit of the nervous system, from which axons grew and relied upon for nutrition; that neurons were discontinuous and formed physical contacts at which communication occurred; and that information flowed across neurons in one direction, from the dendrite toward the axonal terminals (reviewed by Fishman, 1994; Llinas, 2003; Guillery, 2005; Lopez-Munoz et al., 2006). These central tenets are now universally accepted, with caveats (Guillery, 2005), but spawned an acrimonious and dogmatic battle between “reticularists” and “neuronists” that persisted from the late 1870s until the invention of the electron microscope and subsequent visualization of mammalian synapses in the 1950s.^2^

## The Field of Connectomics

**FIGURE 1:**
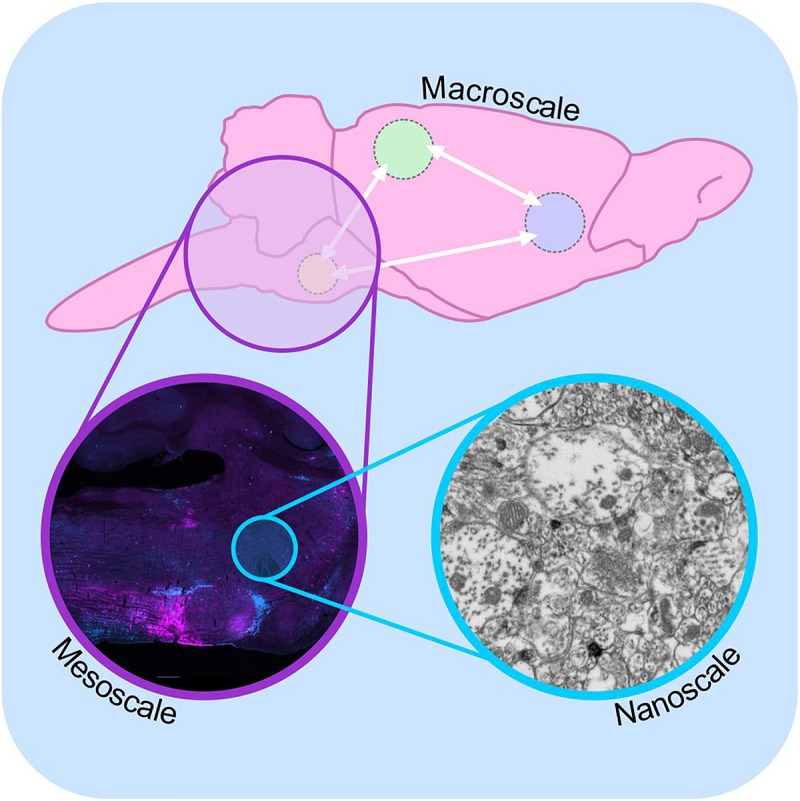
Macro-, meso-, and microscale connectomics. Schematic diagram illustrating connectome analysis at macroscale (top), mesoscale (purple inset), and nanoscale resolutions (blue inset). Macroscale approaches (top) examine communication between regions of the brain on a global level, using approaches such as diffusion-weighted magnetic resonance imaging. Mesoscale connectomics interrogates neural circuitry at the cellular level, employing light microscopy to map the distribution of synaptically linked neurons, in this case monosynaptic inputs to putative sympathetic premotor neurons (see Menuet et al., 2017, modified with permission). Nanoscale connectomics assesses individual synaptic contacts using electron microscopy. Nanoscale image shows electron micrograph of synapses and polysialyic acid immunoreactivity: see Bokiniec et al. (2017).

Since then, generations of neuroscientists have used progressively more selective labeling techniques and more powerful microscopes to reveal the patterns of synaptic connectivity that are thought to underlie the functional properties of neural circuits. This effort has given rise to the field of “connectomics,” a now standalone sub-discipline of neuroscience with the stated aim of understanding the “*structural architecture of nervous system connectivity in all animals at all resolutions*” (reviewed by Hagmann, 2005; Sporns et al., 2005; Swanson and Bota, 2010; Catani et al., 2013). Connectomics generates simplified circuit diagrams at macroscopic (brain-wide), mesoscopic (circuit level) or nanoscopic (synapse level) resolutions that classify neurons in terms of their connectivity to each other (Figure 1: discussed by Branco and Staras, 2009). However, these efforts have been hampered by both the technical complexity involved in accurately identifying synaptic connections and the sheer magnitude of the task: full connectomic reconstruction of the human brain would require the mapping of approximately 86 billion neurons (Azevedo et al., 2009; Herculano-Houzel, 2009) and the identification of the thousands of inputs and outputs that connect each one (Nimchinsky et al., 2002).

As a result of this complexity, the only entire connectomes thus far cataloged belong to tiny animals: the first (nearly) complete map of the entire nervous system of an individual species was made by White et al. (1986) from reconstructed serial electron micrographs of the roundworm *Caenorhabditis elegans* (later completed by Varshney et al., 2011), and more recently larval and adult *Drosophila melanogaster* (Ohyama et al., 2015; Zheng et al., 2018) and the larval sea-squirt *Ciona intestinalis* (Ryan et al., 2016). The “nanoscale” approach used to map these connectomes offers comprehensive knowledge of every neuronal connection, and is well-suited to components of relatively small local circuits such as in the retina, in which the inputs and outputs are understood (Briggman et al., 2011; Denk et al., 2012).

However, ultrastructural strategies are poorly suited to the interrogation of large or dispersed circuits because of the enormous investments of time and infrastructure required to acquire and handle the data (Lichtman and Denk, 2011; Wanner et al., 2015). These technical limitations seem unlikely to be resolved in the foreseeable future; despite recent innovations such as the development of serial block-face and Focused Ion Beam electron microscopy, which have reduced the acquisition time for a cubic millimeter of tissue from ∼18 to ∼1.5 years (Wanner et al., 2015; Xu et al., 2017), obstacles to the analysis and even the storage of high resolution microscopy data remain. For example, the raw dataset for a single human brain would require ∼175 exabytes of storage space, costing 2–8 billion Euros (Mikula, 2016). By comparison, the entire storage capacity of the planet in 2011 was ∼295 exabytes (Hilbert and Lopez, 2011).

These limitations have provided a stimulus for the development of genetically modified viral tracers that can be used to identify components of a given neuronal circuit without requiring direct visualization of synaptic contacts. The major advantages of this approach are its compatibility with light microscopy, greatly reducing the imaging, analysis and storage burden inherent to ultrastructural analysis, and its applicability to mapping networks that are dispersed throughout the brain.

## Anterograde and Retrograde Tracers

**FIGURE 2:**
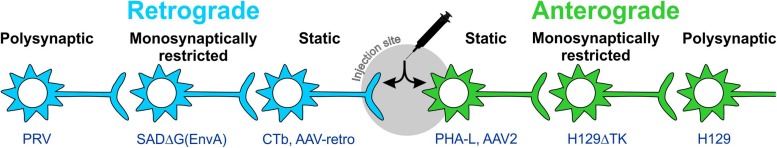
Anterograde and retrograde labeling with static and trans-synaptic tracers. Tracers are categorized as anterograde or retrograde based on their direction of travel within neurons. Anterograde tracers (green) are taken up by neuronal cell bodies at the injection site and travel down the axon to terminal processes. Retrograde tracers (blue) are taken up by terminals and travel back to the cell body. Static tracers remain within the first neurons they enter, while other tracers can spread trans-synaptically and may or may not be monosynaptically restricted. CTb: cholera toxin B subunit; H129ΔTK: tyrosine kinase-deleted H129 herpes; PHA-L: *Phaseolus vulgaris*-leucoagglutinin; PRV: pseudorabies virus; SADΔG(EnvA): glycoprotein-deleted EnvA-pseudotyped rabies.

Early tracing approaches involved the physical or electrical lesion of a region of interest, which rendered the degenerating axons differentially susceptible to impregnation with metallic silver (Hoff, 1932). Although crude, this approach was eventually refined to the point where terminal boutons could be resolved (Glees, 1946; Nauta, 1952; Fink and Heimer, 1967). Visualization of Wallerian degeneration was replaced by approaches that did not require destructive lesions, relying instead on the axonal transport of injected/applied materials from the site of injection to either the cell body (retrograde tracers) or axonal processes (anterograde tracers; Figure 2). The earliest of these tracers were radiolabeled amino acids such as tritiated leucine and proline that were injected into neural tissue then incorporated into polypeptides in the soma and transported to axons and terminal processes where they were identified by autoradiography (Grafstein, 1967; Cowan et al., 1972). These tracers were soon surpassed by materials that could be detected with conventional light microscopy via histological or immunohistochemical (IHC) processing, intrinsic fluorescence, or conjugation with a fluorophore or enzymatically active probe. This variety of tracer constitutes what are now referred to as “conventional tracers” (in contrast to viral tracers). The variety and utility of conventional tracing techniques are broad and have been extensively reviewed elsewhere (Köbbert et al., 2000; Vercelli et al., 2000; Lanciego and Wouterlood, 2011): here, we provide a simple overview of some of the most popular conventional tracers for comparison with the viral tracers discussed in upcoming sections.

### Conventional (Mainly) Retrograde Tracers

The glycoprotein and enzyme horse radish peroxidase (HRP) was found in the early 1970s to be an effective retrograde tracer, having been taken up into neurons non-selectively by passive endocytosis (Kristensson and Olsson, 1971; LaVail and LaVail, 1972; Köbbert et al., 2000; Vercelli et al., 2000). HRP is visualized as it catalyzes, together with hydrogen peroxide, the oxidation of chromogenic substrates such as 3,3′-diaminobenzidine and tetramethylbenzidine. The resultant staining is limited to the cell soma and primary dendrites (Köbbert et al., 2000) and, on its own, HRP demonstrates a relatively low sensitivity due to inefficient uptake by neurons at the injection site. However, conjugation of HRP to the plant lectin wheat germ agglutinin (WGA), itself a neuronal tracer, significantly improved both uptake and transport within neurons (Staines et al., 1980; Köbbert et al., 2000). WGA binds to *N*-acetylglucosamine and the plasma membrane-bound sugar sialic acid, and is rapidly actively transported in both the anterograde and retrograde directions, providing more extensive (but not complete) labeling of the neuron compared to HRP (Schwab et al., 1978; Dumas et al., 1979; Levy et al., 2017). WGA-HRP conjugates are also capable of limited trans-synaptic travel (Goshgarian and Buttry, 2014; Sillitoe, 2016), introducing some ambiguity to the interpretation of data. WGA is now available in a fluorophore-conjugated preparation, eliminating the need for immunohistochemical processing for visualization.

Cholera toxin subunit B (CTb) was introduced as a retrograde tracer in 1977 (Stoeckel et al., 1977; Vercelli et al., 2000). Trojanowski et al. (1981, 1982) then sought to improve the sensitivity of HRP using CTb-HRP conjugates, and found that when the number and detail of labeled neurons was compared, CTb-HRP significantly out-performed free HRP. They attributed this to the GM1 ganglioside (sugar) mediated uptake of CTb in comparison to the non-specific endocytosis of HRP (Trojanowski et al., 1981; Trojanowski et al., 1982; Lanciego and Wouterlood, 2011). Unconjugated CTb can be detected via immunohistochemistry, however, the development of CTb-conjugated fluorophores (Conte et al., 2009a, b) or even magnetically opaque labels visible to MRI (Wu et al., 2011) has extended the scope of CTb. The signal strength of fluorescently conjugated CTb, its rapid transport (2–7 days), low toxicity, and ease of use also makes it suitable for identification of neurons for subsequent electrophysiological recordings *in vitro* (Korim et al., 2014; Bou Farah et al., 2016) or *in vivo* (Yamashita and Petersen, 2016), and has elevated fluorescently conjugated CTb variants as a go-to retrograde tracer for many researchers (Parker et al., 2015; Zhao et al., 2017), although it should be noted that, like most conventional tracers, CTb transport is to some extent bidirectional (Noseda et al., 2010). In our experience, CTb-conjugated fluorophores can suffer quenching when used in conjunction with *in situ* hybridization, and as the fluorophore prevents binding of anti-CTb antibodies, quenched signal cannot be boosted with anti-CTb IHC, so for dual labeling of mRNA and CTb the unconjugated form is preferable.

A number of inorganic tracers are also widely used. Hydroxystilbamidine (FluoroGold^TM^) is a fluorescent inorganic compound directly visible with fluorescence microscopy. Following uptake into nerve terminals by fluid phase endocytosis, FluoroGold^TM^ is transported retrogradely to cell bodies in vesicles and accumulates in the cytoplasm, where it remains detectable for months (Köbbert et al., 2000; Lanciego and Wouterlood, 2011). The intense, bleach-resistant labeling achieved using FluoroGold^TM^, and the availability of anti-FluoroGold^TM^ antibodies for further amplification, has resulted in it becoming the “gold-standard” tracer in rodents, against which newly developed tracers are compared (Lanciego and Wouterlood, 2011; Tervo et al., 2016).

Other noteworthy inorganic fluorescent tracers are Fast Blue, Diamidino Yellow, True Blue and the carbocyanines DiI and DiO (Bentivoglio et al., 1980; Bonhoeffer and Huf, 1980; Kuypers et al., 1980; Puigdellivol-Sanchez et al., 1998; Schofield, 2008; Yu et al., 2015). Although no longer widely used for tracing projections in the central nervous system, they (along with organic tracers such as CTb, WGA, etc.) remain popular choices for the identification of autonomic, motor or sensory innervation of peripheral targets, into which they can be injected in relatively large volumes (Furukawa et al., 2008; Zele et al., 2010; Yu et al., 2015; Pidsudko et al., 2019; Rytel et al., 2019). Interestingly, because they are not dependent on active transport, inorganic dyes may also be used for identification of projections in fixed tissue *post mortem*. Typically applied as crystals to the surface of formalin-fixed tissue blocks, highly lipophilic dyes such as DiI (red) and DiO (green) move evenly throughout cells in both anterograde and retrograde directions via the lipid portion of neuronal membranes, resulting in complete labeling of the soma and dendritic tree (Thanos et al., 1991, 1992; Köbbert et al., 2000; Boon et al., 2019; Trivino-Paredes et al., 2019). However, the lipophilic nature of carbocyanine dyes makes them difficult to use in conjunction with standard IHC protocols that use lipid-solubilizing detergents to facilitate antibody penetration (Elberger and Honig, 1990; Matsubayashi et al., 2008).

Fluorescent latex microspheres 30–90 nm in diameter (Retrobeads^TM^) were recognized as retrograde tracers in the mid-1980s (Katz et al., 1984; Katz and Iarovici, 1990) and, like FluoroGold^TM^, are highly resistant to fading and offer long-term labeling. Although somewhat difficult to use, based on their tendency to clump in the injection pipette, latex beads offer stable fluorescence with minimal diffusion from the injection site, exclusive retrograde travel with minimal entry to undamaged fibers of passage, and are non-toxic to neurons so can be used in long duration experiments (Köbbert et al., 2000; Vercelli et al., 2000; Lanciego and Wouterlood, 2011). Their distinctive bright punctate appearance makes them easy to distinguish, meaning they can be mixed with an anterograde tracer of the same color [e.g., to simultaneously identify retro- and anterograde projections from a single brain region (Turner et al., 2013)].

### Conventional (Mainly) Anterograde Tracers

*Phaseolus vulgaris*-leucoagglutinin (PHA-L) is one of the earliest and most widely used anterograde tracers (Gerfen and Sawchenko, 1984; Köbbert et al., 2000) and, like WGA, PHA-L binds to membrane bound carbohydrates to gain cell entry (in this case *N*-acetyl D-glucosamine and mannose). PHA-L is detected using IHC, revealing detailed cell morphology, including axon terminal branches to the level of terminal boutons (Lanciego and Wouterlood, 2011; Wouterlood et al., 2014). PHA-L requires longer post-injection survival times to achieve transport compared other conventional tracers, typically 10 to 20 days (Vercelli et al., 2000).

Emerging in the mid-1980s, dextran-amines (DAs) were rapidly adopted and remain widely used as conventional axonal tracers (Gimlich and Braun, 1985; Glover et al., 1986; Brandt and Apkarian, 1992; Veenman et al., 1992; Wouterlood et al., 2014). DAs enter injured neurons at the injection site and spread evenly throughout the entire neuron via diffusion, resulting in a Golgilike level of staining detail (Glover et al., 1986; Fritzsch, 1993; Glover, 1995; Köbbert et al., 2000; Lanciego and Wouterlood, 2011; Wouterlood et al., 2014).

Despite the common perception that DAs are preferential anterograde tracers, many studies indicate bidirectional travel (Schmued et al., 1990; Fritzsch, 1993; Glover, 1995; Zhang et al., 2017), including the original description of their axonal transport by Glover et al. (1986). Their retrograde capabilities have been exploited both for conventional tracing (Sivertsen et al., 2014, 2016; Lunde et al., 2019) and for delivery of calcium-sensitive indicators for optical recording of neurons selected by axonal trajectory (O’Donovan et al., 1993; McPherson et al., 1997).

There is a perception that the molecular weight of DA-conjugates contributes to their directional selectivity, with smaller molecules exhibiting superior performance as a retrograde tracer (Reiner et al., 2000; Lanciego and Wouterlood, 2011). However, the influence, if any, of molecular weight on directional specificity is probably overstated, and may instead reflect differences in speed of transport, which is distinctly faster for smaller compounds (Fritzsch, 1993), combined with differences in volume of synaptic terminals compared to cell bodies (Glover, personal communication).

Like CTb, fluorophore-labeled dextran amine variants are now widely used instead of or in addition to biotinylated versions that require histological processing for visualization, and we and others have used tetramethylrhodamine-conjugated dextran for juxtacellular labeling during electrophysiological recordings (Noseda et al., 2010; Dempsey et al., 2015).

### Limitations of Conventional Tracers

Despite their ongoing popularity, the major limitations of conventional tracers are worthy of consideration:

(1) Conventional tracers can be taken up by fibers of passage (Dado et al., 1990; Chen and Aston-Jones, 1995; Conte et al., 2009a), which can lead to incorrect identification of projections. [Notably, canine adenovirus (CAV) can also be taken up by fibers of passage (Schwarz et al., 2015)].
(2) The spread of many conventional tracers around the injection site results in intense and diffuse labeling that may reflect deposition in the extracellular matrix or take-up by neurons or glia. Such non-specific labeling makes it difficult to reliably identify labeled neurons within ∼1 mm of the injection site. Thus the historical use of conventional tracers has probably overemphasized the relative significance of distant inputs/outputs compared to those originating from local interneurons; contemporary connectomic studies indicate that long-distance projections are relatively rare compared to short-distance connections (Oh et al., 2014; Henriksen et al., 2016; van den Heuvel et al., 2016; Dempsey et al., 2017).
(3) Tracer uptake relies predominantly on sugars that are located on the glycocalyx of most, if not all neurons, or on common mechanisms such as endocytosis. Consequently, restricted uptake by functionally or neurochemically/genetically homogeneous neuronal populations is not possible.
(4) The direction of axonal transport is rarely exclusive, which complicates circuit analysis; the archetypal retrograde and anterograde tracers, CTb and BDA respectively, both label axons traveling in the “wrong” direction (Luppi et al., 1987; Schmued et al., 1990; Fritzsch, 1993; Glover, 1995; Angelucci et al., 1996; Noseda et al., 2010; Zhang et al., 2017).

## Viral Tracers

Recombinant viral vectors that drive the expression of fluorescent “reporter” proteins in transduced neurons have been widely adopted by neuroscientists because of their directional specificity, the high (in most cases permanent) levels of reporter expression obtained, and the absence of transduction of fibers of passage (comprehensively reviewed by Callaway, 2005; Luo et al., 2008; Betley and Sternson, 2011; Nassi et al., 2015). Here, we will examine some widely used variants and consider the extent to which they address the limitations of conventional tracers.

Viral tracers may be divided into two distinct classes: static vectors that remain locked within the targeted cell population and essentially function like conventional tracers, which are usually replication-deficient, and vectors that spread through linked circuits via trans-synaptic travel, which are almost always replication-competent. Each class contains vectors that can be used to label selectively in an antero- or retrograde direction.

### Static Viral Tracers

Recombinant vectors derived from human immunodeficiency virus (HIV), herpes simplex virus type 1 (HSV-1), human and canine adenoviridae (Ad), adeno-associated virus (AAV), semliki forest virus, sindbis virus (SIN), and rabies have all been developed as alternatives to conventional chemical tracers (Chamberlin et al., 1998; Furuta et al., 2001; Wickersham et al., 2007a; Junyent and Kremer, 2015; Lerner et al., 2016; Jia et al., 2017; Farmer et al., 2019). A detailed review of the biology of each is beyond the scope of the current article; the following resources provide useful overviews of the most commonly used replication deficient viral vectors (Ad: Akli et al., 1993, AAV: Kuo et al., 1995; Drouin and Agbandje-McKenna, 2013; Junyent and Kremer, 2015, HIV: Lundberg et al., 2008; Murlidharan et al., 2014, HSV-1: Neve, 2012). HIV, HSV, Ad, SIN, and AAV vectors differ in their maximum genetic payload, whether transgenes are integrated into the host genome, and the onset and duration of gene expression, but share common features: the wild-type virus is modified to remove genes required for viral replication and replaced with a genetic sequence that encodes a reporter protein under the control of a ubiquitous (e.g., cytomegalovirus, CaMKII), pan-neuronal (e.g., neuron-specific enolase, synapsin) or cell-type specific (e.g., PRSx8, GAD1) promoter or, if used in transgenic animals (reviewed by Wang et al., 2011), genetically restricted expression systems such as Cre-LoxP or FLP-FRT (Blomer et al., 1997; Hwang et al., 2001; Jakobsson et al., 2003; Schnutgen et al., 2003; Gofflot et al., 2011; Liu et al., 2013; Fenno et al., 2014; Luo et al., 2018). In contrast, glycoprotein-deleted rabies is a static retrograde vector that retains its capacity for replication, but has been modified so that it can no longer spread trans-synaptically (Larsen et al., 2007; Wickersham et al., 2007a). This has the advantage of self-amplification, resulting in very high levels of reporter expression, but results in cytotoxicity within a few weeks of infection and, as transcription is not promoter-dependent, cannot be targeted to particular cell types without further genomic alterations (discussed below). Furthermore, as the rabies genome is RNA based, strategies for selective recombination within subpopulations of infected neurons (e.g., Cre-LoxP) are not possible.

The mode of use of static viral tracers is similar to conventional tracers; vectors are injected in small volumes into the region or organ of interest under anesthesia, and experimental animals are allowed to recover with appropriate post-operative care. Following a period sufficient for transgene translation, the animal is euthanized and perfused with fixative, and the CNS removed for histological processing, which may include IHC amplification of reporter signal, followed by imaging. The major differences compared to conventional tracers lie in the duration of recovery (although reporter expression is visible within 12 h of HSV or rabies injections, protein transcription is typically optimal 10–20 days after transduction by AAV and lentiviral vectors), the volume of injectate, which is typically larger for conventional tracers (300–1000 nl vs. 20–100 nl for CNS injections, 1–5 μl for peripheral injections into target organs), and the level of biological containment required. When injected into the periphery, a further consideration is the age of the animal; in our experience peripheral injections of rabies and HSV-1 vectors do not result in detectable neuronal labeling when administered to mice older than post-natal day 7, perhaps reflecting the immaturity of the innate and adaptive immune responses in the early post-natal period.

Like conventional tracers, cell entry is mediated via interactions between glycans or proteins expressed on the cell membrane and components of the viral vector (Lykken et al., 2018). The tendency of a given vector to label in an anterograde or retrograde direction therefore reflects the cellular distribution of cognate receptors to its particular surface proteins; binding with receptors expressed on the soma results in anterograde labeling (e.g., AAV serotype 2, 5, 7, 8, and rh.10, human adenovirus), whereas binding with receptors preferentially expressed at the axon terminal results in retrograde labeling (e.g., HSV-1, rabies and canine adenovirus) (Frampton et al., 2005; Berges et al., 2007; Salinas et al., 2009; Penrod et al., 2015; Castle et al., 2016; Hirschberg et al., 2017; Sathiyamoorthy et al., 2017; Farmer et al., 2019). Note that viral titer may also play a role in the direction of travel; human adenovirus becomes a retrograde tracer at high titers as the recruited immune response limits their efficacy (Howorth et al., 2009). Perhaps unsurprisingly, variation of viral surface proteins by natural selection or human manipulation can alter their affinity for cellular binding partners, changing the tropism for different cell types (e.g., neuronal versus non-neuronal) or different neuronal compartments (e.g., pre-synaptic versus post-synaptic) (Kanaan et al., 2017; Lykken et al., 2018). The diverse capsid sequences found in the dozen or so naturally occurring primate AAVs that have been developed as vectors for gene transfer exhibit dramatically different tropisms for different cell types (reviewed by Drouin and Agbandje-McKenna, 2013; Castle et al., 2016) and hosts (Watakabe et al., 2015). When injected into the rodent central nervous system, most result in anterograde labeling, but the AAV1, 5 and 9 serotypes exhibit both anterograde and retrograde transport, with the degree of retrograde tropism varying according to the region studied and construct used (Rothermel et al., 2013; Castle et al., 2016). Altered directional tropism can also be conferred by pseudotyping viral vectors with chimeric envelope proteins from other viruses; this approach has been used with success to switch between anterograde and retrograde tropism in HIV-1 and vesicular stomatitis virus vectors, and to generate rabies variants that can gain cellular access at the soma instead of the axon terminal (Blomer et al., 1997; Wickersham et al., 2007b; Kato et al., 2011; Beier et al., 2011, 2013).

Many different viruses have been developed as potential vehicles for gene delivery, but in recent years AAV vectors have emerged as front-runners (Kanaan et al., 2017). Although the maximum size of the AAV payload is limited compared to lentiviral, adenoviral and HSV vectors, they are easy to work with, have low toxicity and immunogenicity, and result in rapid (∼14 days) and permanent transgene expression in post-mitotic cells such as neurons (reviewed by Chamberlin et al., 1998; Betley and Sternson, 2011; Sun and Schaffer, 2018). These properties have made AAV vectors attractive candidates in gene therapy for cancer, metabolic, and neurological diseases (Weinberg et al., 2013; O’Connor and Boulis, 2015; Santiago-Ortiz and Schaffer, 2016; Grimm and Buning, 2017), stimulating investment in AAV production and targeting technologies that are likely to further accelerate their development. AAV capsids have been manipulated to improve their transduction ability for both gene therapy and neuroanatomical applications (Tervo et al., 2016; Chan et al., 2017). Of particular relevance to the neuroscientist is the recent development of a synthetic AAV capsid that drives retrograde transduction with particularly high efficiency and selectivity (Tervo et al., 2016). This capsid, known as AAV-retro, results in transduction profiles that closely resemble conventional retrograde tracers such as FluoroGold^TM^ (Tervo et al., 2016), is compatible with small injection volumes, and is not associated with significant toxicity (Sun et al., 2019). These features, and the fact that it can be made in any viral production facility that produces conventional AAV vectors, indicate that the popularity of AAVs will continue to increase.

The AAV-retro variant, and other synthetic AAV capsids, were made using *in vivo* directed evolution, a process in which error-prone PCR is used to introduce mutations to the *cap* gene (Drouin and Agbandje-McKenna, 2013). Mutant viral particles are then injected into organoids or animal models and the tissue of interest is harvested, from which AAV variants are isolated and sequenced to identify mutations with the desired tropism (Kotterman and Schaffer, 2014; Sun and Schaffer, 2018). In producing AAV-retro, AAV variants with different *cap* mutations were injected into either the substantia nigra or the cerebellar cortex and harvested from the striatum or inferior olive, respectively, revealing candidate capsid sequences with high selectivity for retrograde transport (Tervo et al., 2016). Given the many dozens of unstudied capsid subtypes that lie within the 13 major groups of primate AAV (Gao et al., 2004), the many thousands of potential capsids that lie within putative ancestral primate AAV libraries (Santiago-Ortiz et al., 2015), and the perhaps millions of capsid variants yet to be isolated and characterized from non-primate AAVs (Smith et al., 2016), it seems likely that AAV capsids with useful tropisms will continue to be discovered and developed for neuroscience applications. This may even lead to development of capsid variants with selectivity for particular neuronal cell types, bypassing the need for recombinase-driving transgenic animals or cell-type specific promoters, the Achilles’ heel of AAV due to its small payload size (although the selectivity of synthetic AAV promoters is also rapidly improving: see Jüttner et al., 2019), with obvious benefits to both research and gene therapy applications.

### Identification of Synaptic Contacts

Irrespective of whether anterograde labeling is achieved by conventional tracers or vector-mediated reporter expression, the identification of terminal appositions under light microscopy remains a challenge because of their small size (Burette et al., 2015). The development of vectors that selectively label presynaptic terminals (e.g., synaptophysin-GFP or synaptophysin-mRuby) makes disambiguation of terminals from axons and fibers easier (Li et al., 2010; Oh et al., 2014; Beier et al., 2015; Lerner et al., 2015), but the fact remains that <50% of “close appositions” constitute functional synapses (Pilowsky et al., 1992; Descarries and Mechawar, 2000; da Costa and Martin, 2011), and so their presence needs to be interpreted with caution. In recent years a number of elegant approaches have been developed to circumvent this limitation, the ultimate of which is GRASP (GFP Reconstitution Across Synaptic Partners), a dual vector system where each vector drives the expression of one half of a GFP-derived dimer that only becomes fluorescent when both halves bind. When presynaptic neurons are targeted with one and post-synaptic neurons with the other, the only place at which the two components can become physically close enough to bind is the synaptic cleft, and so GFP fluorescence denotes synaptic contact (Feinberg et al., 2008; Kim et al., 2011). The interested reader is directed to recent reviews that considers alternative methods for synaptic complementation (Wickersham and Feinberg, 2012; Luo et al., 2018).

## Trans-Synaptic Circuit Tracing

Replication-deficient viral vectors that drive reporter expression are ideal anterograde tracers, because they can target genetically restricted cells in circumscribed brain regions and result in unambiguous labeling of the entire neuron and/or its synaptic terminals. However, for the reasons outlined above, such fine detail is not easy to extract across whole brains and, moreover, anterograde labeling experiments are generally conducted in an effort to determine the post-synaptic targets of a given population of neurons, not to map the distribution or number of synaptic contacts arising from them. For the purposes of identifying post-synaptic targets, a better solution would be a tracer that labels the entire post-synaptic cell, which can be unambiguously and rapidly detected using light microscopy under relatively low magnification instead of the high-resolution methods required to reliably detect individual synapses. One approach that can achieve this exploits the trans-synaptic trafficking of Cre recombinase that occurs when expressed at high levels by *replication-deficient* viral vectors. Zingg et al. (2017) found that AAV-mediated Cre recombinase expressed in presynaptic neurons could drive Cre-dependent transgene expression in post-synaptic neurons, and that the degree of post-synaptic transgene expression, which presumably reflects the degree of Cre recombinase trafficking, varies depending on AAV serotype. This provides a useful tool for identification and manipulation of post-synaptic neurons, although the approach is somewhat confounded by the partial retrograde tropism of the AAV1 and AAV9 serotypes (Rothermel et al., 2013; Castle et al., 2016), which coincidentally produced the highest Cre recombinase trafficking (Zingg et al., 2017), leading to ambiguity over whether tagged neurons represent post- or pre-synaptic network components.

Another strategy that avoids having to identify individual synapses is to infect target populations with a *replication-competent* neurotrophic virus and to trace circuit components via the trans-synaptic infection of viral progeny (comprehensively reviewed by Nassi et al., 2015). This approach, initially developed using wild-type viruses that exhibit selectively retrograde (rabies, pseudorabies) or anterograde tropism (HSV-1 strain H129) (Strack et al., 1989b; Zemanick et al., 1991; Card and Enquist, 1995; Ugolini, 1995; Aston-Jones and Card, 2000; Kelly and Strick, 2000), represents a significant advance over static tracers in the sense that labeling can be safely interpreted as indicating synaptic contact. However, tracing using intact replication-competent viruses suffers two major drawbacks: first, it is never clear whether connections are mono- or polysynaptic, because the replicating virus continues to propagate along the synaptic hierarchy. Second, wild-type replication-competent viruses cannot be used to selectively target groups of neurons embedded in the CNS; direct injection of these vectors gives rise to non-selective infection, and so their use is best restricted to circuits that begin (i.e., sensory) or end (i.e., motor) in the periphery.

### Anterograde Trans-Synaptic Tracing via HSV-1 Strain H129

Investigators have attempted to sidestep the drawbacks listed above by modifying viral constructs to alter their tropisms (so that target populations of neurons can be selectively infected following central injection), by comparing the results of viral and conventional tracing studies (to gain insight into which projections are likely to represent monosynaptic connections), and by performing detailed time-course studies to map the likely hierarchy of neuronal connections. All of these approaches have been performed with H129, a strain of HSV-1 that was originally isolated from a patient who died from acute necrotizing encephalitis (Dix et al., 1983) and is notable for its predominantly anterograde direction of trans-synaptic travel (Archin and Atherton, 2002; Wojaczynski et al., 2015). H129 has been used for the mapping of neural circuits for over 20 years (Sun et al., 1996; Garner and LaVail, 1999; Labetoulle et al., 2000); it exhibits rapid replication and spread, with reporter-driving variants producing detectable fluorescence at the site of primary infection within 24 h of innoculation. In our hands, H129 produces detectable trans-synaptic labeling of visual pathways within 48 h of intraocular injection, and the progression of labeling suggests another synapse is “jumped” every 48 h thereafter, although signal strength is variable and benefits from IHC amplification.

As infection progresses, H129 causes cytopathic changes in cell morphology and, if left unchecked, eventual death of the animal, imposing ethical and practical constraints on the duration of experiments (Lo and Anderson, 2011; McGovern et al., 2012b, Wojaczynski et al., 2015). Inoculated rodents begin to show signs of neurological disease that may include hemiparesis, ataxia and drastic weight loss, which varies in its onset time, severity, and prevalence depending on the site of initial infection (Archin and Atherton, 2002; Rinaman and Schwartz, 2004; McGovern et al., 2012a). Interestingly, a significant proportion of rodents inoculated with H129 never develop any detectable infection: in our hands intraocular infection fails to “take” in almost half of cases, similar to the 36% failure rate reported by Lo and Anderson (2011), who also found a surprisingly high failure rate following direct brain injection (18%). The transient nature of the primary viral infection and host defense mechanisms may contribute to variability in uptake and differences in reported strength of labeling.

**FIGURE 3:**
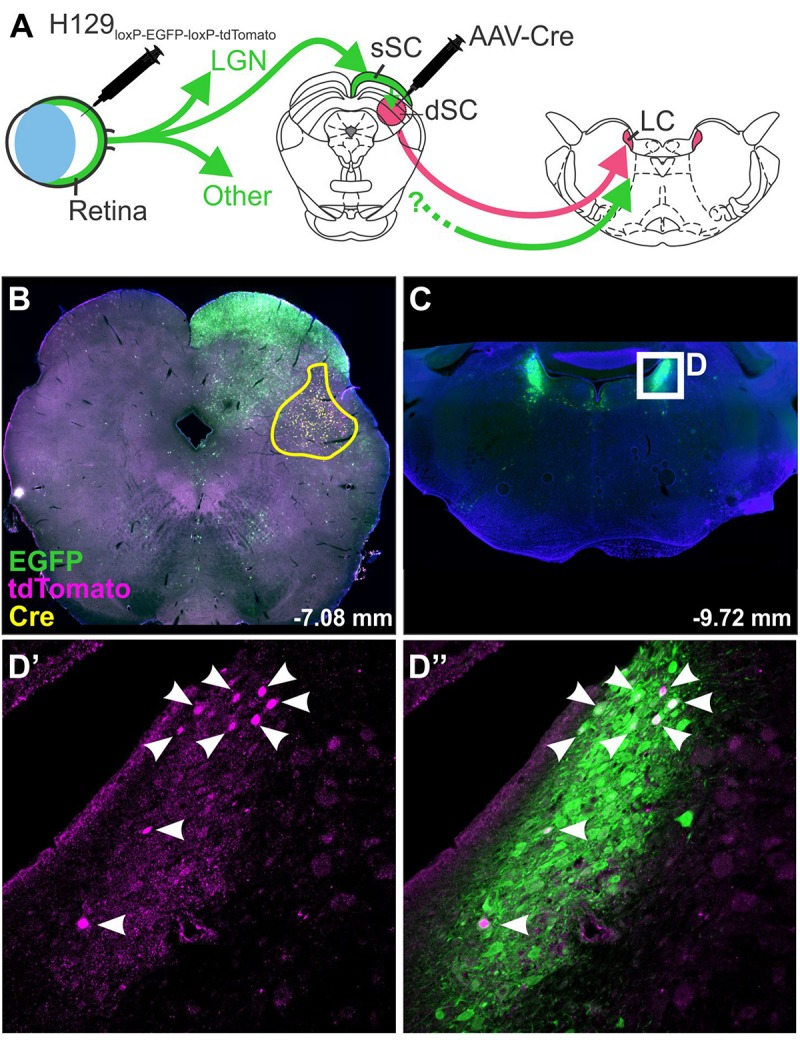
H129 tracing of a polysynaptic pathway. Demonstration of H129 infection of a polysynaptic pathway from the retina, through the deep superior colliculus (dSC), to the locus coeruleus (LC). **(A)** Intravitreal injections of H129-HCMV-loxP-EGFP-HCMVpA-loxP-tdTomato-SV40pA initially infected retinal ganglion cells, driving EGFP expression in retinorecipient nuclei and downstream neurons. When AAV-Cre was injected into the dSC prior to H129 infection (yellow boundary in **B**) the genomes of H129 virions passing through the dSC were cleaved, resulting in tdTomato expression in post-synaptic neurons. Red neurons in downstream regions, such as LC (Low power micrograph in **C**, magnified view in **D’**), can therefore be interpreted as being part of a pathway passing through the dSC. Co-expression of tdTomato and EGFP was common (see merged image, **D”**) and may indicate incomplete H129 cleavage or multiple pathways converging on the same region. LGN: lateral geniculate nucleus; sSC: superficial layer of the superior colliculus.

The specificity of H129 for tracing subpopulations of neurons has been improved by modification of the viral genome: Lo and Anderson (2011) produced a H129 strain dependent on Cre recombinase for replication and transcription of a red reporter. This was achieved by the insertion of a floxed STOP cassette upstream of the tdTomato and TK genes (the TK gene being necessary for replication). Infection of Cre-containing neurons therefore results in permanent removal of the STOP cassette, restoration of viral replication and transcription of the reporter gene, whereas initial infection is unable to take hold in neurons that do not synthesize Cre recombinase. Reporter expression therefore indicates that a neuron either contains Cre recombinase or lies downstream from a Cre-containing neuron, allowing the identification of post-synaptic circuit components. A similar strategy was devised by McGovern et al. (2015b), in which the H129 genome was modified to encode a floxed GFP lying upstream of a red reporter gene (H129-HCMV-loxP-EGFP-HCMVpA-loxP-tdTomato-SV40pA, Figure 3). In this case the GFP gene is expressed in naïve neurons but is excised by Cre, resulting in a switch in reporter color in Cre-synthesizing neurons and their (polysynaptic) downstream partners. These modifications allow H129 to be targeted to subpopulations of central neurons, allowing identification of connected circuits, but these variants are still compromised by ambiguity regarding the number of synapses that link labeled neurons to the originally targeted population and the systemic illness that results from unrestricted central infection. These limitations have been recently overcome by deletion of a gene required for viral replication, tyrosine kinase (*TK*), which arrests the spread of H129. When supplied by a helper vector *in trans*, *TK* transiently restores the ability of H129 to replicate and to spread into monosynaptically connected neurons. As these do not contain *TK*, the H129 becomes trapped and so viral reporter expression can be safely interpreted as denoting monosynaptic spread (Zeng et al., 2017; Li et al., 2018).

### Retrograde Poly-Synaptic Viral Tracers

Rabies and pseudorabies virus (PRV, a member of the alphaherpes virus family) were developed as retrograde trans-synaptic tracers in the latter decades of the 20th century (Strack et al., 1989a; Ugolini, 1995). PRV, HSV-1 and other alphaherpes viridae rely on interactions between the viral envelope glycoprotein complex and cell surface receptors to gain intracellular access (reviewed by Frampton et al., 2005; Sathiyamoorthy et al., 2017); in neuronal tissue, nectin-1, a synaptic adhesion molecule that is predominantly expressed on the presynaptic membrane and colocalizes well with synaptophysin, is the primary mediator of cell entry and infection, without which little neuronal infection occurs (Geraghty et al., 1998; Campadelli-Fiume et al., 2000; Mizoguchi et al., 2002; Takai et al., 2008). In addition to the nectin-1- pathway, it is understood that direct cell-to-cell transfer can occur via non-synaptic mechanisms, and that direction of transport may be bi-directional, particularly in wild-type PRV strains, although this confound is reduced in the PRV-Bartha laboratory strain (Card et al., 1998, 1990, 1991, 1999; Pickard et al., 2002; Ugolini, 2011). As a result, PRV experiments are often limited to short incubations and low viral doses in an attempt to reduce the incidence of non-synaptic release of viral particles via cytotoxic cell death.

PRV spreads rapidly, causing observable transgene expression within 6 h *in vitro*, 1 day *in vivo*, and demonstrating trans-synaptic travel within 2 days *in vivo* (Card et al., 1990; Kobiler et al., 2010). The speed and directional selectivity of PRV has made it the historical tracer of choice for both peripheral and central tracing studies (Martin and Dolivo, 1983; Strack et al., 1989a; Jansen et al., 1995; Smith et al., 1998; DeFalco et al., 2001) although, due to its polysynaptic nature, PRV poses the same challenge as H129 – the differentiation of mono- and polysynaptic connections. As with H129, time-course analyses have sought to address this issue, as well as co-injection of PRV with conventional tracers to identify regions sequentially labeled along polysynaptic pathways (Card et al., 1990; Smith et al., 1998; Kim et al., 1999; Aston-Jones and Card, 2000; DeFalco et al., 2001; Card and Enquist, 2014). Replicating PRV viruses cause neuronal damage and ultimately death of the animal within 5 to 6 days following peripheral injections and 4 days following intracerebral injections (Ugolini et al., 1987; Card et al., 1990, 1992; Card and Enquist, 2014; Oyibo et al., 2014). A less virulent strain has been developed in which the only immediate early gene of PRV, IE80, has been deleted (“IE80-null PRV,” Oyibo et al., 2014). Neurons infected with IE80-null PRV have been reported to maintain physiological properties similar to non-infected neurons up to 6 months post infection, with injected mice surviving the same period of time, demonstrating a promising reduction in toxicity.

As with H129 (McGovern et al., 2015a), strains of PRV encoding different transgenes have been used in dual-tracing experiments in which the aim is to assess whether two populations of neurons receive input from a common, third region (Jansen et al., 1995; Banfield et al., 2003). For example, Jansen et al. (1995) used two strains of PRV-Bartha encoding different viral antigens, one encoding the viral glycoprotein gC, and the other encoding β-galactosidase. One strain was injected into the stellate ganglion, while the second was injected into the adrenal gland and IHC used to identify single- and double-labeled neurons. While dual tracing studies appear deceptively simple, a number of important factors must be taken into consideration when selecting the viral vectors to use. The core issue is “superinfection inhibition”; the limited capacity of a single neuron to be infected by more than one virus (Kobiler et al., 2010; Card and Enquist, 2014). Alterations to the viral genome, including the transgene expressed and the location of its insertion, influence the virulence and efficiency of expression, which may place a vector at a disadvantage compared to a “fitter” strain (Kobiler et al., 2010; Card and Enquist, 2014), and so isogenic strains of viruses should be used to minimize these differences (Smith et al., 2000; Banfield et al., 2003). The speed of travel and therefore time of arrival of a virus to the population of interest is also critical; Banfield et al. (2003) compared the dual-infection of rat dorsal root ganglion neurons *in vitro* by PRV152 and PRV614 introduced asynchronously. They found that when the vectors were added simultaneously, almost all neurons were double-labeled, but that a delay of 2 h between injections reduced double-labeling by ∼70%, and that a 4-h delay resulted in almost none. Furthermore, there is a limit to the number of copies of a transgene that a neuron can produce: Kobiler et al. (2010) injected three isogenic strains of PRV that expressed the “Brainbow” cassette and, based on the combinations of fluorophores expressed, estimated that no more than seven genomes could be expressed in each cell. Therefore, vectors should be chosen that are as closely matched as possible for genomic sequence, virulence, speed of transduction and transgene expression, and should arrive at the common region of interest at as close a timepoint as possible.

### Retrograde Trans-Synaptic Tracing via Glycoprotein-Deleted Rabies

The evolution of the TK-deleted H129 virus parallels the development of glycoprotein-deleted rabies, the first virus to be developed as a genetically restricted monosynaptic tracer (reviewed by Luo et al., 2008, 2018; Callaway and Luo, 2015). It was developed from the Street Alabama, Dufferin B19 (SADB19) strain of rabies, a virus isolated from a symptomatic dog in the 1930s and maintained on cultured rodent tissue for decades thereafter, over which time it became rodent-specific, attenuated, and incapable of horizontal or vertical transmission (Vos et al., 1999; Beckert et al., 2009). These features make recombinant SADB19 not only a safer option as a research tool, but also led to its development as a live oral vaccine for wild foxes across central Europe, with over 50 million doses airdropped since the 1980s (Geue et al., 2008).

Genetically modified SADB19 has provided a new generation of tracing tools that avoid the shortcomings of classic replication-competent neurotrophic viruses. At the heart of this technology is the key role the rabies glycoprotein plays in the infection and exclusively retrograde trans-synaptic spread of rabies, which occurs through still-undefined mechanisms (Lentz et al., 1982; Mebatsion et al., 1996; Etessami et al., 2000; Schnell et al., 2010; Ugolini, 2010). Deletion of the glycoprotein gene renders the virus, so-called SADΔG, incapable of spreading from one neuron to another but does not affect its capacity for replication (Mebatsion et al., 1996; Etessami et al., 2000; Wickersham et al., 2007a), meaning that SADΔG variants becomes trapped inside infected neurons.

The key insight made by Wickersham et al. (2007b) is that SADΔG’s ability to trans-synaptically migrate can be transiently restored by expression of the rabies glycoprotein in infected neurons *in trans* (Marshel et al., 2010). For example, when motoneurons are induced to express rabies glycoprotein using an AAV vector, subsequent infection of those neurons by intramuscular injection of SADΔG results in retrograde infection of monosynaptic input neurons in the spinal cord and brainstem (Esposito et al., 2014).

**FIGURE 4:**
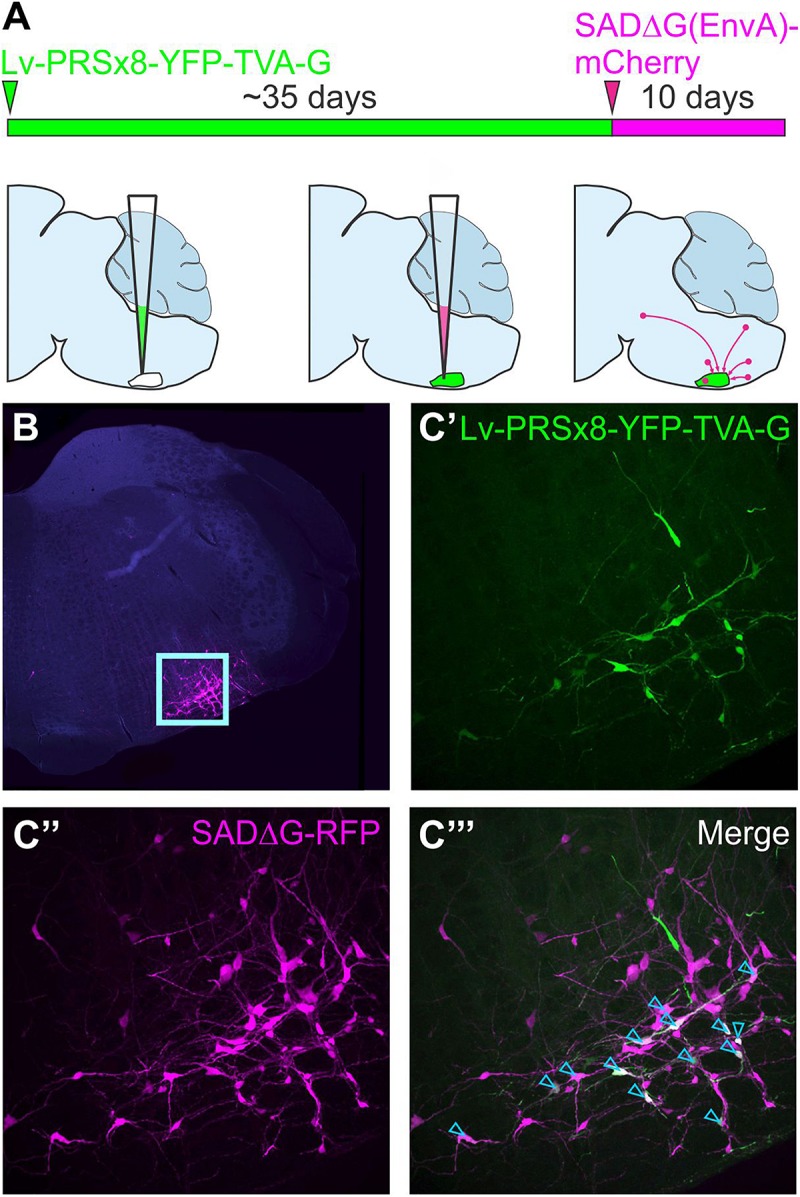
Monosynaptically restricted retrograde tracing using glycoprotein-deleted rabies. **(A)** Experimental strategy: the starting point is co-expression of genes that encode TVA and the rabies glycoprotein in target neurons, in this case delivered by a lentiviral vector that targets putative sympathetic premotor neurons in the ventrolateral medulla (Lv-PRSx8-YFP-TVA-G). Subsequent infection of TVA/G-expressing neurons by SADΔG(EnvA) microinjection leads to trans-synaptic infection of pre-synaptic (“input”) neurons. Panel **(B)** shows low power micrograph with infected neurons concentrated in the ventrolateral medulla (boxed region, enlarged in C) “Seed” neurons may be distinguished from “input” neurons by co-expression of the reporters contained in the lentiviral **(C’)** and rabies constructs **(C”)**, which appear white in the merged image (**C”’**, denoted by blue arrowheads). Modified with permission from Menuet et al. (2017).

This approach was further refined by restricting initial access of SADΔG to targeted populations of neurons by pseudotyping glycoprotein-deleted rabies with the envelope protein of a virus that is unable to infect naïve mammalian neurons, avian sarcoma and leukosis virus (EnvA). Infection by pseudotyped SADΔG, notated as SADΔG(EnvA), can be controlled by selective expression of its cognate receptor, TVA, on target neurons (Wickersham et al., 2007b; Marshel et al., 2010). The starting point for monosynaptic retrograde tracing strategies that use SADΔG(EnvA) can be defined by co-expression of the genes that encode TVA and the rabies glycoprotein in target neurons, often referred to as “seed” or “starter” neurons. Direct CNS injection of SADΔG(EnvA) selectively infects seed neurons at the injection site, wherein virions replicate, incorporate the glycoprotein, and spread to monosynaptically connected pre-synaptic (“input”) neurons. To distinguish input neurons from seed neurons, a reporter that differs in color from that contained in the SADΔG genome is by convention included within the TVA/glycoprotein cassette (Figure 4).

In theory, this approach could be used to identify monosynaptic inputs to any population in which it is possible to selectively drive TVA and the rabies glycoprotein. Investigators from diverse branches of neuroscience have used innovative approaches to limit seeding to their populations of interest, using transgenic mice (Takatoh et al., 2013), lentiviral, AAV or HSV-1 viral vectors (Brennand et al., 2011; Liu et al., 2013; Yonehara et al., 2013; Dempsey et al., 2017; Menuet et al., 2017), and even *in vivo* transfection of single functionally identified neurons (Rancz et al., 2011; Velez-Fort et al., 2014; Wertz et al., 2015).

SADΔG(EnvA) represents a current state-of-the-art technique for interrogation of circuit structure; its promise is reflected by its rapid adoption by diverse branches of neuroscience, high citation rates of studies that use it, and the continued investment in its refinement. However, it is not without its limitations: first, the proportion of monosynaptic input neurons labeled by SADΔG(EnvA) is low and subject to variability, with the ratio of input neurons per starter neuron ranging from less than 10 to several hundred (Rancz et al., 2011; Velez-Fort et al., 2014; Stornetta et al., 2016). Although some of this variability reflects actual differences in the numbers of afferent neurons that converge upon different cell types (Velez-Fort et al., 2014), as discussed at length by Callaway and Luo (2015), the strength of glycoprotein/TVA expression on the target neuron, the titer of SADΔG(EnvA) (and therefore initial number of replicating virions within starter cells), and the duration of the experiment also interact to determine labeling efficiency. Efforts to improve labeling efficiency have focussed on modification of the rabies glycoprotein, resulting in a codon-optimized chimera of the SADB19 and Pasteur G glycoproteins known as “oG” (Kim et al., 2016), or generation of glycoprotein-deleted variants of other rabies strains (Reardon et al., 2016), both of which have improved the efficiency of the original system.

A second caveat to the use of glycoprotein-deleted rabies variants lies in uncertainty regarding potential biases in its tropism. Although validation of trans-synaptic infection of excitatory and inhibitory neurons was included in the initial description of this system (Wickersham et al., 2007b), the potential for differential selectivity according to proximity or phenotype has been raised by a number of investigators (Callaway and Luo, 2015; Reardon et al., 2016). Recent work by Sun et al. (2019) has confirmed these concerns, showing differential labeling of neurons by AAV-retro, SADΔG and conventional tracers, as well as by polysynaptic strains of rabies and pseudorabies, which they speculate reflects variable expression of cognate receptors to viral proteins in different neuronal populations.

A third weakness of the SADΔG system is its neurotoxicity, which is low compared to H129 but nonetheless results in microglial infiltration, gene dysregulation, and cell death within a few weeks of infection (Wickersham et al., 2007a; Sun et al., 2019), and is a major barrier to its integration with functional tools. Again, the development of glycoprotein-deleted versions of other rabies strains has been reported to reduce toxicity (Reardon et al., 2016), and much has been made of a self-inactivating form that clears infected neurons of histotoxic elements shortly after infection (Ciabatti et al., 2017), although a recent early report by Wickersham et al. throws the validity of this approach into doubt (preprint by Matsuyama et al., 2019). Perhaps more promising is the development of so-called “double-deletion rabies” variants (SADΔGL), in which the “L” gene responsible for transcription and replication of the viral genome is deleted in addition to that of the glycoprotein, resulting in a virus with negligible gene transcription and toxicity but which can nonetheless drive expression of recombinase proteins at levels sufficient to drive site-specific recombination in transgenic animals (Chatterjee et al., 2018). Although the recent work by Chatterjee et al. (2018) supports the suitability of SADΔGL as an alternative to AAV-retro, the applicability of this system as a monosynaptically restricted tracer is still unknown.

Finally, in contrast to PRV and H129, the cellular mechanisms responsible for the transmission of rabies are yet to be comprehensively delineated, resulting in some skepticism regarding its supposed retrograde trans-synaptic exclusivity (eloquently summarized in a recent blog by Svoboda, 2019). The strongest evidence in favor of the exclusivity of this mechanism is broad coherence between the results of rabies tracing, ultrastructural, and electrophysiology studies (considered extensively by Ugolini, 2011), and in particular the absence of rabies virions from the extracellular space (which would indicate non-synaptic transmission). However, as pointed out by Svoboda, without a complete understanding of the rabies life cycle it remains difficult to account for discrepancies in connectivity schemes suggested by the results of viral tracing and electrophysiological mapping studies, and so some caution should be exercised in the interpretation of these data.

## Imaging, Analysis and Presentation of Connectomic Data

The repurposing of viral vectors as instruments for scientific research has transformed the field of neuroanatomy in less than 20 years. The mass production of viral vectors by commercial “vector cores” has lowered economic barriers to their adoption by researchers, while the development of technologies that permit high-throughput manipulation of viral genomes and selection of useful traits, such as directed evolution and genetic barcoding, has made it easier than ever for vector biologists to prototype new viral tools. These factors, coupled with the scientific merit of the approach, will drive innovation in this space for the foreseeable future.

It is therefore surprising that the technologies used by researchers to capture, analyze and present anatomical data have changed so little over the same period: with few exceptions, researchers continue to present their data in terms of the numbers of labeled neurons that lie within each region of the brain, averaged across replicates and estimated by comparing images of histological sections to plates from a 2-D reference atlas. As we have argued before (Dempsey et al., 2017), expressing rich datasets in such crude terms precludes their independent analysis by other researchers, while potential future reclassification of brain taxonomy threatens the shelf-life of the data. Moreover, it is a laborious and error-prone process, because accuracy is contingent on cutting histological sections in perfect alignment with the reference atlas while maintaining correct ordering and orientation. This task sounds straightforward but is hard to achieve in reality – it took George Paxinos over 50 attempts to get a perfect example for the original Paxinos and Watson rat brain atlas (G. Paxinos, personal communication) – and small deviations from the orthogonal make correct alignment with the reference atlas impossible; for example, if a section through the widest part of the rat brain is cut at 5° mediolateral from true coronal alignment, the lateral border of the cortex on one side of the brain will lie 1.4 mm rostral to the other.

How should this problem be addressed? In our view, it will not be resolved by improving imaging technologies *per se* – if one’s objective is to simply map populations of labeled neurons, then improvements in optical resolution are unlikely to help much (although the lowered cost, improved speed and spectral sensitivity of modern microscopes present benefits of their own). Nor are recently developed tissue processing techniques that permit continuous imaging of thick volumes of brain likely to provide a simple solution (Chung et al., 2013; Renier et al., 2014; Tomer et al., 2014): although useful for imaging small volumes of brain, they are slow, labor- and resource-intensive, and inherently difficult to align to a reference atlas. Instead, a new generation of tools is required that can automate and standardize the analysis of tracing data by automatically registering histological tissue into 3-d volumetric brain atlases, extracting the locations of labeled neurons and expressing them in Cartesian co-ordinates as well as identifying the regions in which they reside. This development would simplify analysis, reduce variability, and permit the sharing and independent analysis of connectivity data. Although a number of investigators have highlighted the importance of this mission (Osten and Margrie, 2013; Renier et al., 2016; Furth et al., 2018; Puchades et al., 2019), to date the tools available are hard to use and far from intuitive.

## Conclusion

Connectomic data will not, by itself, explain how brains work: connectivity is but one parameter in an overlapping spectrum of classifications used by neuroscientists to bracket neurons. However, when combined with electrophysiological properties, synaptic strength, neurotransmitter content, receptor expression and developmental lineage, connectivity data may provide clues to the processes that underlie the function of specific brain circuits and provide insights into the general rules that underlie brain circuit formation, growth and plasticity (Rockland, 2015).

Nevertheless, controversy about the utility of extensive connectomic data remains unresolved (Carandini, 2012; Denk et al., 2012; Rockland, 2015; Jonas and Kording, 2017) (interested readers are also encouraged to watch the debates between Anthony Movshon and Sebastian Seung hosted by Neuwrite at Columbia University in 2012^3^ and between Anthony Movshon and Moritz Helmstaedter, held as part of the Cognitive Neuroscience Society annual meeting in 2016^4^). Similar skepticism characterized the early years of the Human Genome Project: the scientific value of sequencing the whole human genome, the exploratory nature of the Project, the seemingly insurmountable technical challenges and high cost projections were a cause for concern for scientists, politicians and tax-payers alike (reviewed by Gannett, 2016). However, the completion of the Human Genome Project not only dramatically advanced sequencing techniques (e.g., personal genealogical genotyping is now commercially viable), but also accelerated the incorporation of genetic technologies to biology at large and spawned gene therapy (Lander, 2011). Whether the discoveries that emerge from the contemporary neuroscience equivalents of the Human Genome Project will lead to analogous translational technologies remains to be seen, but the connectomic movement has already transformed neuroscience.

## Author Contributions

All authors contributed to the planning, discussion, and drafting of the manuscript.

## Conflict of Interest Statement

SL was a Ph.D. student at the time of writing. He is now an employee of the Olympus Australia. He has approved the submission of the manuscript but has not provided any material input since becoming an Olympus Australia employee. The remaining authors declare that the research was conducted in the absence of any commercial or financial relationships that could be construed as a potential conflict of interest.

## Footnotes

1 Ironically, despite having already been discredited as a serious science by then, phrenology contributed to the general acceptance of this view by fostering the notion that individual variation in discrete brain functions are reflected by skull topography (summarized by Gross, 2009).

2 Opponents to the neuronist school have been characterized as subscribing to a chaotic and somewhat absurd view of the brain. Guillery (2005) makes a compelling case for reassessment of this historical debate, highlighting discoveries that were made after the description of the physical structure of the synapse (e.g., gap junctions, axo-axonic contacts) that fundamentally undermine some tenets of the neuron theory. Guillery argues that both models were ultimately wrong, and that a less dogmatic post-neuronist view is more appropriate.

2 https://youtu.be/fRHzkRqGf-g

4 https://youtu.be/uSbNRyY2QH0
