# Corrigendum: A Student's Guide to Neural Circuit Tracing

**DOI:** 10.3389/fnins.2020.00177

**Published:** 2020-03-10

**Authors:** Christine Saleeba, Bowen Dempsey, Sheng Le, Ann Goodchild, Simon McMullan

**Affiliations:** ^1^Neurobiology of Vital Systems Node, Faculty of Medicine and Health Sciences, Macquarie University, Sydney, NSW, Australia; ^2^The School of Physiology, Pharmacology and Neuroscience, University of Bristol, Bristol, United Kingdom; ^3^CNRS, Hindbrain Integrative Neurobiology Laboratory, Neuroscience Paris-Saclay Institute (Neuro-PSI), Université Paris-Saclay, Gif-sur-Yvette, France

**Keywords:** neuroanatomy, viral tracers, anterograde tracer, retrograde tracers, synaptic contacts, connectome analysis

In the original article, there was an error: one section of our review considers reagents traditionally considered to be anterograde tracers (i.e., fluorescent or antigenic substances that are taken up by neuronal cell bodies at the site of application and transported to the synaptic terminals). The original text read:

Emerging in the mid-1980s, dextran-based tracers, particularly biotinylated dextran amine (BDA), were rapidly adopted and remain one of the most widely used conventional anterograde tracers (Glover et al., 1986; Brandt and Apkarian, 1992; Veenman et al., 1992; Wouterlood et al., 2014). BDA enters injured neurons at the injection site, undergoes rapid anterograde transport and spreads evenly throughout the entire neuron, resulting in a Golgi-like level of staining detail (Köbbert et al., 2000; Lanciego and Wouterlood, 2011; Wouterlood et al., 2014). Interestingly, while 10 kDa BDA travels mostly anterogradely, the 3 kDa form is a retrograde tracer (Reiner et al., 2000; Lanciego and Wouterlood, 2011). Like CTb, fluorophore-labeled dextran amine variants are now widely used instead of biotinylated versions that require histological processing for visualization, and a number of authors have used tetramethylrhodamine-conjugated dextran for juxtacellular labeling during electrophysiological recordings (Noseda et al., 2010; Dempsey et al., 2015).

**Limitations of Conventional Tracers**

Despite their ongoing popularity, the major limitations of conventional tracers are worthy of consideration:

1. Conventional tracers are taken up by fibers of passage (Dado et al., 1990; Chen and Aston-Jones, 1995; Conte et al., 2009), which can lead to incorrect identification of projections. [Notably, canine adenovirus (CAV) can also be taken up by fibers of passage (Schwarz et al., 2015)].
2. The spread of many conventional tracers around the injection site results in intense and diffuse labeling that may reflect deposition in the extracellular matrix or take-up by neurons or glia. Such non-specific labeling makes it difficult to reliably identify labeled neurons within ~1 mm of the injection site. Thus the historical use of conventional tracers has probably overemphasized the relative significance of distant inputs/outputs compared to those originating from local interneurons; contemporary connectomic studies indicate that long-distance projections are relatively rare compared to short-distance connections (Oh et al., 2014; Henriksen et al., 2016; van den Heuvel et al., 2016; Dempsey et al., 2017).
3. Tracer uptake relies predominantly on sugars that are located on the glycocalyx of most, if not all neurons, or on common mechanisms such as endocytosis. Consequently, restricted uptake by functionally or neurochemically/genetically homogeneous neuronal populations is not possible.
4. The direction of axonal transport is often not exclusive, which complicates circuit analysis; for example, CTb, perhaps the mostly widely used “retrograde” tracer, is also an efficient anterograde tracer (Luppi et al., 1987; Angelucci et al., 1996; Noseda et al., 2010).

The authors were contacted by Professor Joel Glover, who first described the use of dextran amines as neuronal tracers in the 1980s and expressed concern that we had inadvertently perpetuated a myth regarding the directional sensitivity of these tracers.

The amendment to the article clarifies the bidirectional nature of dextran amine transport.

A correction has been made to the Anterograde and Retrograde Tracers section, subsection Conventional (Mainly) Anterograde Tracers;

“Emerging in the mid-1980s, dextran-amines (DAs) were rapidly adopted and remain widely used as conventional axonal tracers (Gimlich and Braun, 1985; Glover et al., 1986; Brandt and Apkarian, 1992; Veenman et al., 1992; Wouterlood et al., 2014). DAs enter injured neurons at the injection site and spread evenly throughout the entire neuron via diffusion, resulting in a Golgi-like level of staining detail (Glover et al., 1986; Fritzsch, 1993; Glover, 1995; Köbbert et al., 2000; Lanciego and Wouterlood, 2011; Wouterlood et al., 2014).

Despite the common perception that DAs are preferential anterograde tracers, many studies indicate bidirectional travel (Schmued et al., 1990; Fritzsch, 1993; Glover, 1995; Zhang et al., 2017), including the original description of their axonal transport by Glover et al. (1986). Their retrograde capabilities have been exploited both for conventional tracing (Sivertsen et al., 2014, 2016; Lunde et al., 2019) and for delivery of calcium-sensitive indicators for optical recording of neurons selected by axonal trajectory (O'Donovan et al., 1993; McPherson et al., 1997).

There is a perception that the molecular weight of DA-conjugates contributes to their directional selectivity, with smaller molecules exhibiting superior performance as a retrograde tracer (Reiner et al., 2000; Lanciego and Wouterlood, 2011). However, the influence, if any, of molecular weight on directional specificity is probably overstated, and may instead reflect differences in speed of transport, which is distinctly faster for smaller compounds (Fritzsch, 1993), combined with differences in volume of synaptic terminals compared to cell bodies (Joel C Glover, personal communication).

Like CTb, fluorophore-labeled dextran amine variants are now widely used instead of or in addition to biotinylated versions that require histological processing for visualization, and we and others have used tetramethylrhodamine-conjugated dextran for juxtacellular labeling during electrophysiological recordings (Noseda et al., 2010; Dempsey et al., 2015).

**Limitations of Conventional Tracers**

Despite their ongoing popularity, the major limitations of conventional tracers are worthy of consideration:

1. Conventional tracers can be taken up by fibers of passage (Dado et al., 1990; Chen and Aston-Jones, 1995; Conte et al., 2009), which can lead to incorrect identification of projections. [Notably, canine adenovirus (CAV) can also be taken up by fibers of passage (Schwarz et al., 2015)].
2. The spread of many conventional tracers around the injection site results in intense and diffuse labeling that may reflect deposition in the extracellular matrix or take-up by neurons or glia. Such non-specific labeling makes it difficult to reliably identify labeled neurons within ~1 mm of the injection site. Thus the historical use of conventional tracers has probably overemphasized the relative significance of distant inputs/outputs compared to those originating from local interneurons; contemporary connectomic studies indicate that long-distance projections are relatively rare compared to short-distance connections (Oh et al., 2014; Henriksen et al., 2016; van den Heuvel et al., 2016; Dempsey et al., 2017).
3. Tracer uptake relies predominantly on sugars that are located on the glycocalyx of most, if not all neurons, or on common mechanisms such as endocytosis. Consequently, restricted uptake by functionally or neurochemically/genetically homogeneous neuronal populations is not possible.
4. The direction of axonal transport is rarely exclusive, which complicates circuit analysis; the archetypal retrograde and anterograde tracers, CTb and BDA respectively, both label axons traveling in the “wrong” direction (Luppi et al., 1987; Schmued et al., 1990; Fritzsch, 1993; Glover, 1995; Angelucci et al., 1996; Noseda et al., 2010; Zhang et al., 2017).”

The authors apologize for this error and state that this does not change the scientific conclusions of the article in any way. The original article has been updated.
